# Is there a role for routine intraoperative cholangiogram in diagnosing CBD stones in patients with normal liver function tests? A prospective study

**DOI:** 10.1515/iss-2023-0059

**Published:** 2024-03-08

**Authors:** Yi Ping Lim, Voon Meng Leow, Jun Kit Koong, Manisekar Subramaniam

**Affiliations:** Department of Surgery, University Malaya, Kuala Lumpur, Malaysia; USMMC, Bertam, Kepala Batas, USM, Penang, Malaysia; Hepatobiliary Unit, Department of General Surgery, 124937 Hospital Sultanah Bahiyah, Alor Setar, Kedah, Malaysia

**Keywords:** choledocholithiasis, cholangiography, cholecystectomy, common bile duct gallstones, liver function tests

## Abstract

**Objectives:**

Cholecystectomy with or without intraoperative cholangiogram (IOC) is an accepted treatment for cholelithiasis. Up to 11.6 % of cholecystectomies have incidental common bile duct (CBD) stones on IOC and 25.3 % of undiagnosed CBD stones will develop life-threatening complications. These will require additional intervention after primary cholecystectomy, further straining the healthcare system. We seek to examine the role of IOC in patients with normal LFTs by evaluating its predictive values, intending to treat undiagnosed CBD stones and therefore ameliorate these issues.

**Methods:**

All patients who underwent cholecystectomies with normal LFTs from October 2019 to December 2020 were prospectively enrolled. IOC was done, ERCPs were performed for filling defects and documented as “true positive” if ERCP was congruent with the IOC. “False positives” were recorded if ERCP was negative. “True negative” was assigned to normal IOC and LFT after 2 weeks of follow-up. Those with abnormal LFTs were subjected to ERCP and documented as “false negative”. Sensitivity, specificity, and predictive values were calculated.

**Results:**

A total of 180 patients were analysed. IOC showed a specificity of 85.5 % and a NPV of 88.1 % with an AUC of 73.7 %. The positive predictive value and sensitivity were 56.5 and 61.9 % respectively.

**Conclusions:**

Routine IOC is a specific diagnostic tool with good negative predictive value. It is useful to exclude the presence of CBD stones when LFT is normal. It does not significantly prolong the length of hospitalization or duration of the cholecystectomy hence reducing the incidence of undetected retained stones and preventing its complications effectively.

## Introduction

Cholelithiasis is common and may be one of the most difficult diseases to treat if complicated, causing a substantial burden on healthcare resources worldwide [1]. Approximately 35 % of symptomatic gallstones require cholecystectomy [2] and about 10–20 % of patients with gallstones are found to have common bile duct (CBD) stones [3]. Laparoscopic cholecystectomy with or without intraoperative cholangiogram (IOC) is the gold standard treatment for gallstones [4]. However, preoperative identification of asymptomatic CBD stones remains a challenge. Literature suggests up to 11.6 % of patients undergoing cholecystectomy will have silent CBD stones detected on intraoperative cholangiogram [5].

The main aim of performing IOC is to identify CBD stones and aberrant biliary tree anatomy present in 35 % of patients [6]. Although IOC has been widely performed, the routine use of IOC remains controversial [7]. Initial recommendations advocated routine IOC to reduce the incidence of symptomatic CBD stones, identify aberrant anatomy, and hence reduce the incidence of bile duct injuries [8]. Advocates of routine IOC suggest that routine IOC was associated with better recognition of bile duct injuries intraoperatively which influences the success of repair and long-term complications [9, 10]. A systemic review relating to bile duct injuries reported a protective effect of IOC on bile duct injuries and recommended its routine use [11]. Selective IOCs have a 4 times higher rate of missing bile duct injuries and CBD stones compared to routine IOCs. This translates into higher readmissions, costs, and mortality. On the contrary, some studies also suggest that routine use of IOC leads to neither a reduced risk of CBD injuries nor better outcomes [12, 13]. Taking into account the cost of the procedure, increased operative time, and a false positivity rate of 1.6 %, its routine use was not justified [14]. It overestimates the diagnosis of CBD stones, subjects patients to unnecessary instrumentation of the bile duct, and increases its associated morbidity and mortality risks [15, 16]. Despite the years of an ongoing debate regarding routine or selective IOC, the results from a recent meta-analysis in 2021 by Rystedt et al., confirm the use of routine IOC as it reduces the risk and prevents BDI during cholecystectomy. This systemic review was done to evaluate the potential benefits, risks, and cost-effectiveness of shifting from routine to selective IOC. The health economic model generated from this analysis demonstrates that routine IOC is a potentially cost-effective intervention compared to selective IOC on demand [17].

While the natural history of CBD stones is not well described, the GallRiks study [18] suggests that these CBD stones should be removed to reduce the risk of complications that develop over time. 25.3 % of patients with retained CBD stones post-cholecystectomy develop complications such as pancreatitis, cholangitis, and bile duct obstruction as compared to 12.7 % who had CBD stones removed [8]. The incidence of bile leak post cholecystectomy is between 1.4 and 7% [19], and the most common cause is a cystic stump leak. An estimated 20–35 % of cystic stump leaks are caused by retained CBD stones due to an increase in intraluminal pressure [20]. Other examples of these complications are cystic stump breakdown post cholecystectomy due to residual stones and impaction. The European Society of Gastrointestinal Endoscopy (ESGE) Guidelines [21] recommends that a conservative approach should only be considered in patients where the risks of CBD stone retrieval are higher than leaving these stones *in situ*. Hence, all detected CBD stones should be dealt with whether surgically or endoscopically.

IOC is not regularly done in patients with normal LFTs. In the literature, large-scale studies on routine IOC in detecting CBD stones included a significant proportion of high-risk patients in which the need for routine IOC is warranted [4]. According to the American Society of Gastrointestinal Endoscopy (ASGE) 2019 and ESGE guidelines [21] on risk group classification criteria, high-risk patients are defined as patients with cholangitis or CBD stones on ultrasonography (USG)/cross-sectional imaging or total bilirubin>4 mg/dL and dilated CBD on USG/cross-sectional imaging. These high-risk features justify the need for routine IOC in these groups of patients. On the other hand, the ESGE guidelines’ definition of low risk is patients with normal LFTs and USG findings.

In this study, incorporating the definition of low-risk patients according to the ESGE guidelines, we focused on this group of patients in which preoperative LFTs were normal and USG showed no dilated CBD or stones. The primary objective was to examine the predictive values of routine IOC during cholecystectomies in detecting CBD stones in this group of low-risk patients and whether IOCs should be routinely performed for them. To determine the outcome of our primary objective, a post-operative ERCP was performed for patients with raised ALP and GGT after 2 weeks and for patients with filling defects detected on IOC. The purpose of performing an ERCP in this group of patients is to confirm the presence of stones by firstly, re-interpreting the cholangiogram and secondly, by therapeutically removing the detected stone by trawling during ERCP, objectively confirms the presence or absence of CBD stones seen during IOC. Our secondary objectives were to determine the clinical impact of IOC in terms of length of hospitalization and duration of surgery.

## Materials and methods

This is a prospective cohort study that includes all patients with normal LFT who underwent cholecystectomies between October 2019 to December 2020 (15-month duration) in a single tertiary centre in Hospital Sultanah Bahiyah, Kedah, Malaysia. Routine IOC was performed and interpreted.

**Figure 1:**
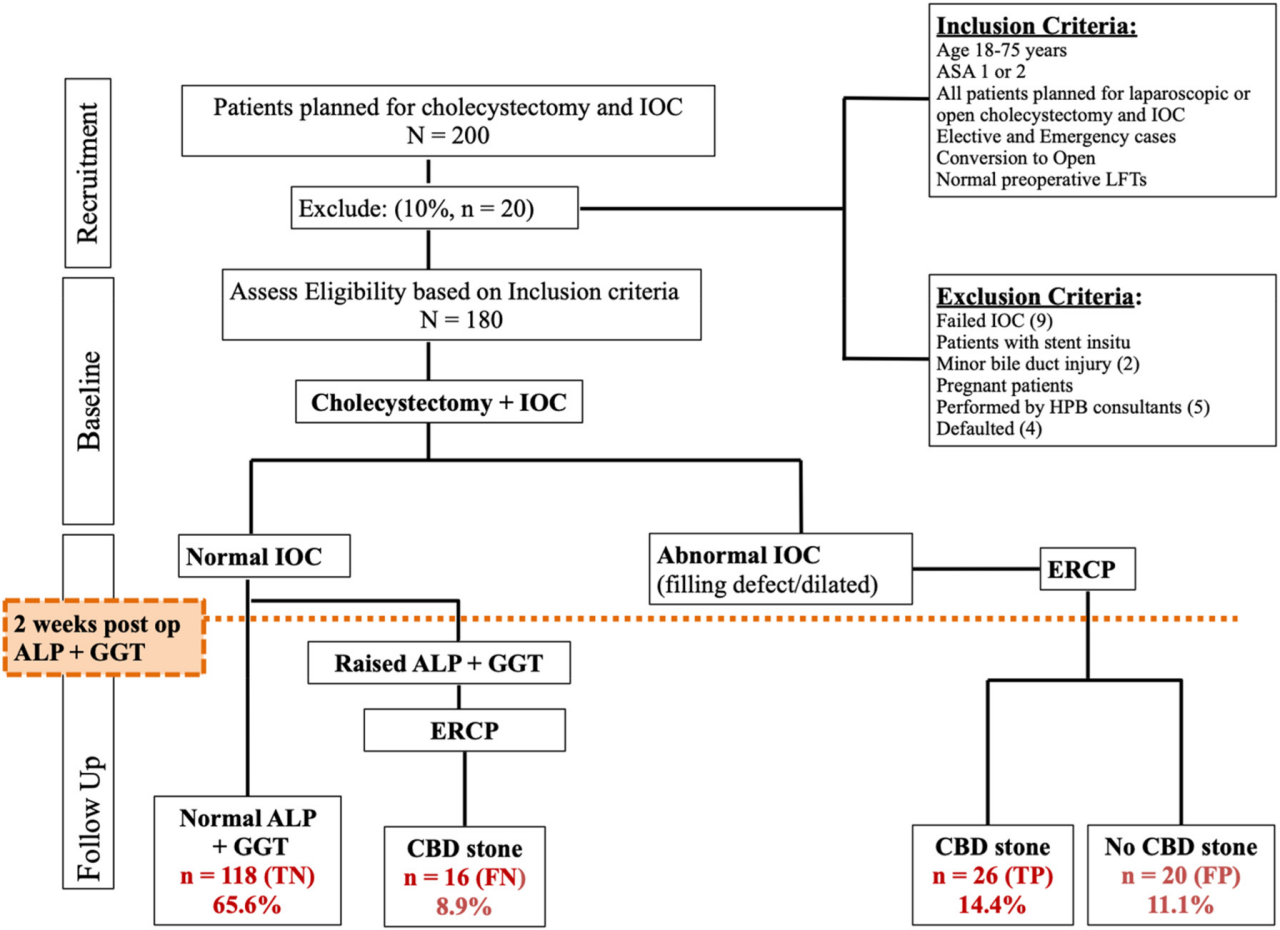
Strobe flow diagram. ALP, Alkaline phosphatase; GGT, Gamma-glutamyltransferase.

IOC was done for all 180 patients, interpreted intraoperatively and findings were documented. The inclusion criteria consist of patients aged between 18 and 75 years old, ASA score of one or two, both laparoscopic or open cholecystectomies, elective or emergency cases, and normal preoperative LFT. Patients with failed IOC, preexisting stent *in situ*, previous severe gallstone pancreatitis or cholangitis, dilated CBD or CBD stones on USG, bile duct injuries, pregnant patients, and cholecystectomies performed by Hepatobiliary consultants were excluded from this study Figure 1.

### Intraoperative cholangiography

A standardized transcystic IOC is performed, with dynamic real-time interpretation of the fluoroscopic cholangiogram using a mobile C-arm Image Intensifier X-ray equipment and 10–40 mls of Diodrast (contrasted medium).

### Criteria for validating IOC

Positive IOC is defined as filling defects detected on IOC. These patients are subjected to ERCP.

True positive IOC: positive IOC with ERCP confirmed CBD stones.

False positive IOC: positive IOC with no CBD stones on ERCP.

Normal IOC is defined as no filling defects on IOC.

True negative IOC: normal IOC with normal post-operative LFT.

False negative IOC: normal IOC with raised post-operative LFT. Following this, a diagnostic and therapeutic ERCP confirmed the presence of CBD stones.

Post-operatively, patients were followed up in the clinic twice; once at 2 weeks for the post-operative LFT and a second time at 3 months to review their general well-being and histopathological examination results of the gallbladder.

### Statistical methods

#### Sample size calculation

Using the sample size formula, *Z*-score table (*Z*=1.96) we calculated a sample size of 180 patients.

Sample size formula:

$$\frac{Z^{2}\left(p\right)\left(1-p\right)}{c^{2}}$$

This was calculated with a 5 % margin of error, a 95 % confidence interval, and a study power of 90 %. The prevalence rate used was 11.6 %.

Statistical analyses were performed with IBM SPSS version 26. Variables collected included demographics, preoperative diagnoses, method of cholecystectomies, IOC findings, duration of surgery, length of stay, ERCP findings, and post-operative LFTs.

Sensitivity, specificity, and predictive values were calculated according to these definitions:

Sensitivity: the percentage of true CBD stones in which IOC indicated CBD stones and confirmed by ERCP.

Specificity: the percentage of true absence of CBD stones (normal CBD) in which had normal IOC.

Positive predictive value: percentage of CBD stones on IOC in which true stones were verified on ERCP.

Negative predictive value: percentage of normal IOC in which no CBD stones were detected postoperatively, reflected by normal LFT.

For our secondary objectives, Chi-squared and Mann-Whitney-U test were used to calculate the statistical significance of the duration of surgery and length of hospitalization respectively with a pre-set p-value of < 0.05.

Ethical Approval was obtained from the National Medical Research Register (NMRR) of Malaysia via the Medical Research and Ethics Committee (MREC), Ministry of Health Malaysia on the seventh of October 2019, NMRR-19-2639-50247.

## Results

A total of two hundred patients underwent cholecystectomy with IOC during the duration of this study. 20 patients (n=10 %) were excluded from the study based on the exclusion criteria. The baseline characteristics of the patients were studied as demonstrated in Table 1. The mean age of the patients studied was 50 years old, with a female: male ratio of 2:1. Majority were of Malay ethnicity (84.4 %) followed by Chinese ethnicity (8.3 %) and Indian ethnicity (4.4 %). 11 patients had a history of mild gallstone pancreatitis and 23 patients had previous mild cholangitis (as defined by the Tokyo 18 Guidelines). The latter had preoperative diagnostic and therapeutic ERCPs done for which existing stones were removed endoscopically. Patients who were stented preoperatively were not included in this study.

**Table 1:** Clinicopathological data (n=180).

Variables	Mean (%)
Age, years	50.1 (15.21)
Gender	
*Male*	61 (33.9)
*Female*	119 (66.1)
Ethnicity	
*Malay*	152 (84.4)
*Chinese*	15 (8.3)
*Indian*	8 (4.4)
Co-morbidities	
*Hypertension*	71 (39.4)
*Diabetes mellitus*	52 (28.9)
*Dyslipidaemia*	43 (23.9)
*Chronic kidney disease*	3 (1.7)
*Liver cirrhosis*	1 (0.6)
*Cerebral vascular accident*	5 (2.8)
*Ischaemic heart disease*	7 (4.4)
*Asthma*	9 (5.0)
*Others*	28 (15.6)
*None*	71 (39.4)
Past surgical history	
*Yes*	40 (22.2)
*No*	140 (77.8)
History of pancreatitis	
*Yes*	11 (6.1)
*No*	169 (93.9)
History of cholangitis	
*Yes*	23 (12.8)
*No*	157 (87.2)
Type of operation	
*Elective (Daycare)*	47 (26.1)
*Elective (Non-daycare)*	71 (39.4)
*Emergency*	62 (34.4)
Method of operation	
*Laparoscopic cholecystectomy with IOC*	127 (70.5)
*Laparoscopic converted to open cholecystectomy with IOC*	20 (11.1)
*OC + IOC*	33 (18.3)
Duration of surgery, mins	
*Less than 60*	91 (50.6)
*61 to 120*	87 (48.3)
*More than 120*	2 (1.1)

The most common indication for cholecystectomy was gallstones (66.7 %), followed by acute cholecystitis (17.7 %), gallbladder empyema (14.4 %), and Mirrizi Syndrome (1.1 %). Preoperatively, 76.6 % (n=138) of patients were diagnosed by ultrasonography and 20.5 % (n=37) by CT scan while 2.7 % (n=5) were diagnosed by both preoperative USG and CT scan.

From the 180 IOCs, 25.6 % (n=46) were found to have filling defects, and the presence of choledocholithiasis was confirmed by post-operative ERCP. 74.4 % (n=134) did not have choledocholithiasis. 65.6 % (n=118) had normal IOC and LFT on follow-up whilst 8.9 % (n=16) of patients had abnormal LFT on follow up and ERCP was performed.

Performing IOCs in patients with normal LFT in a daycare, elective, or emergency setting, does not significantly prolong the duration of surgery (p=0.069, p=0.478, p=0.471, respectively). In an elective setting, the length of hospital stay is not significantly affected by performing IOCs that were straightforward or difficult to interpret (p=0.085) (Table 2).

**Table 2:** Association between duration and length of stay stratified by type of operation among patients (n=180).

Type of operation	Duration of surgery (mins)	Mean (±SD)	p-Value ^a^
Daycare (n=46)	Less than 60	50.5 (8.6)	0.069
	60 to 120	93.9 (15.0)	
	More than 120	0 (0.0)	
Elective (n=72)	Less than 60	47.9 (9.0)	0.478
	60 to 120	93.8 (16.1)	
	More than 120	0 (0.0)	
Emergency (n=62)	Less than 60	48.2 (9.3)	0.471
	60 to 120	91.6 (14.8)	
	More than 120	155.0 (7.1)	

Length of stay (days)		
Type of operation	Median (IQR)	p-Value ^b^
Daycare (n=46)	1.0 (0–2.0)	0.002
Elective (n=72)	1.0 (0.3–3.0)	0.085
Emergency (n=62)	3.0 (1.0–4.0)	0.003

^a^Chi-squared test was applied, ^b^Mann-Whitney-U test was applied, Level of significant set at<0.05.

**Figure 2:**
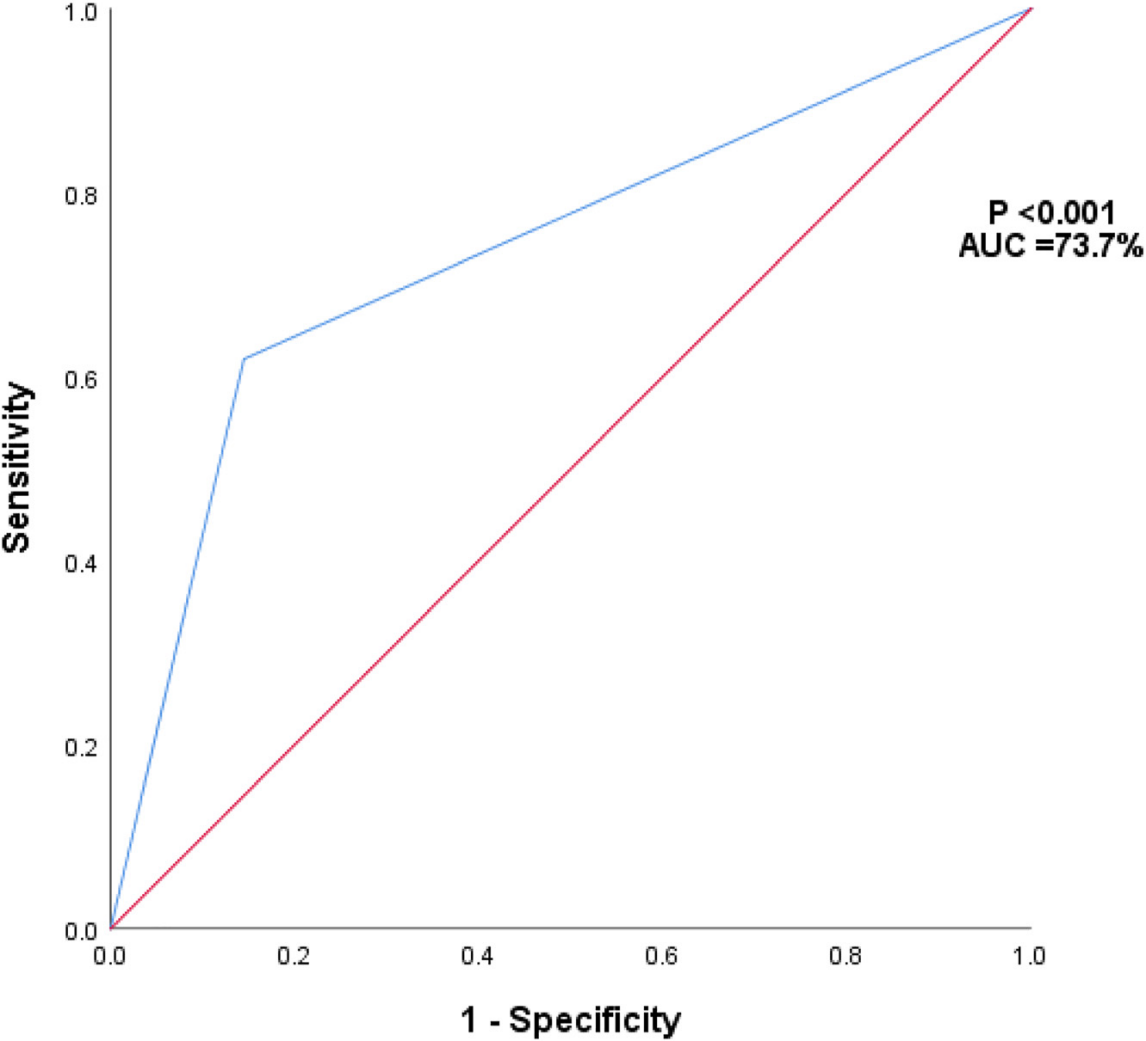
ROC curve and area under ROC curve of IOC predicting CBD stones amongst patients with normal liver function tests (n=180).

This study demonstrated that IOC performed for choledocholithiasis in normal LFTs has a specificity of 85.5 % and a negative predictive value of 88.1 % with an area under the curve of 73.7 % (Figure 2). The positive predictive value and sensitivity were 56.5 and 61.9 %, respectively, with a diagnostic odds ratio of 0.3 (Table 3).

**Table 3:** Agreement between findings of CBD stones on IOC and presence of CBDs on ERCP, and agreement between normal IOC and missed CBD stones on postoperative ERCP combined with post-operative ALP + GGT amongst patients with normal liver function tests (n=180).

CBD stones on IOC	True CBD stones on ERCP Frequency, %		Se	Sp	PPV	NPV	DOR
	Yes	No					
Yes	TP: 26 (14.4)	FP: 20 (11.1)	61.9	85.5	56.5	88.1	0.3
No	FN: 16 (8.9)	TN: 118 (65.6)					
Total	42	138					

TP, True positive; FP, False positive; TN, True negative; FN, False negative; Se, Sensitivity; Sp, Specificity; PPV, Positive predictive value; NPV, Negative predictive value; DOR, Diagnostic odd ratio; Se, Sp, PPV and NPV presented in percentage.

## Discussion

From this study, we demonstrated that the routine use of IOC during cholecystectomy in patients with normal LFT plays a fundamental role in the detection of intraoperative CBD stones. The detection rate of CBD stones was 25.6 % slightly higher than the detection rate of 20 % reported in previous studies [3], [4], [5]. All the detected CBD stones were successfully extracted on post-operative ERCP. There were no major bile duct injuries, post-operative readmissions, or deaths reported during this study, which further emphasizes that IOC is an accepted and safe procedure for the management of CBD stones.

Although some may dispute that low-risk and asymptomatic gallstones should be left alone given the natural history of gallstones, the GREPCO study has shown that the natural history of gallstones is less benign than generally considered [22]. In a local study, a conversion rate of 22 % from asymptomatic to symptomatic over an average of 4.02 years of follow-up was demonstrated [23]. This further supports that CBD stones should be dealt with early especially in patients with normal LFTs when the detection rate is lower than those in the high-risk group (previous severe pancreatitis, cholangitis, or cholecystitis).

The routine use of IOC has always been a debatable issue. Advocates of IOC have cited several advantages such as the diagnosis of unsuspected stones, and delineation of biliary anatomy to detect and reduce biliary injury risk; however, many have abandoned this practice. It has been shown that up to 25.3 % of CBD stones may develop future complications such as pancreatitis, cholangitis, and bile duct obstruction as compared to 12.7 % when CBD stones are removed [18]. The IOC specificity of 85.5 % in our study corresponds to the gold standard MRCP specificity of 83.3–95 % [24], [25], [26]. As the NPV of MRCPs is low in meta-analyses, an NPV of 88.1 % from our study using IOC, adds value in our clinical setting in detecting these CBD stones. From our results, IOCs have a high specificity of 85.5 % which is comparable to that of an MRCP, an NPV of 88.1 %, and an AUC of 73.7 %. This demonstrates that IOC is a good diagnostic test to perform to exclude the presence of CBD stones in patients with normal LFTs. In terms of post-operative management, a positive IOC is as valuable as a negative IOC finding. When confronted with symptomatic patients on post-operative follow-up, negative IOC assessments and permanent intraoperative records are pertinent in the management of these patients.

In our study, a combination of serum ALP and GGT was used to screen for CBD stones postoperatively. This is more convenient, reliable, and cost-effective in screening for biliary obstruction as compared to performing an MRCP which is expensive [27]. Other imaging modalities are deemed inappropriate for screening purposes; USG is operator-dependent, with an accuracy ranging from 20 to 80 % [28, 29] while ERCP or EUS are invasive methods. Although MRCP is the gold standard and non-invasive, it is not cost-effective to subject all patients to postoperative MRCPs. Studies on the accuracy of MRCP in diagnosing CBD stones have also shown a low NPV (44.4–50 %); with a sensitivity of 73.3–80 %, specificity of 83.3–95 %, and PPV of 88.2–98 % [24], [25], [26, 30]. The sensitivity (93.5 %) and specificity (85.1 %) of a combination of ALP and GGT in comparison to ALP (sensitivity 65.1 %, specificity 59.8 %) and GGT (sensitivity 90.8 %, specificity 83.6 %) alone, respectively, renders this combination a reliable screening test for CBD stones [27], hence this was used in this study.

A false positive rate of 11.1 % was higher than the quoted figure of 5 % [5], and this could be attributed to emergency cholecystectomies which were included in this study. A subgroup analysis performed showed that the false positive rate in an emergency setting was 16.1 % compared to a rate of 8.5 % when performed electively. The majority of the difficult IOC interpretation was encountered during emergencies, and this is likely one of the causes that contributed to the higher false positive rates. Looking at the true positive value of 14.4 % (n=26) and taking into account 25.3 % of retained CBD stones who develop complications, the number needed to treat (NNT) is 30. Further studies need to be done to determine the detection rates of retained CBD stones with routine IOC and how it correlates with the 2.3–5% incidence of retained CBD stones post-cholecystectomy [31, 32].

A total of 20 patients (11.1 %) in the false positive group were subjected to ERCP postoperatively but showed no CBD stones. This raises concerns about unnecessary radiation exposure from fluoroscopy during the ERCP as it poses a long-term risk for cancers. However, the lifetime attribute risk of cancer is small, with an incidence of 4.08 and 16.81 per million procedures in diagnostic and therapeutic ERCP respectively [33]. Radiation doses are also dependent on the fluoroscopy time which is subsequently dependent on a few variables such as diagnosis, type of procedure, the procedure setting (diagnostic or therapeutic), endoscopic skills, and patient factors [34]. In our study, all patients in the false positive group underwent a diagnostic ERCP**.** The ESGE guidelines report a mean value of entrance skin dose (ESD) during ERCP ranging between 55 and 347 mGy, and these values are approximately three times higher for therapeutic ERCP compared to diagnostic ERCP [35]. The amount of radiation absorbed by the body differs between diagnostic ERCP and therapeutic ERCP with mean effective doses of 3–6 millisieverts (mSv) for diagnostic ERCP and 12–20 mSv for therapeutic ERCP [36]. In comparison, the mean effective dose for a CT abdomen ranges between 6.8 and 22.4 mSv per procedure [37, 38].

The duration of hospitalization following cholecystectomy is prolonged in cases with an initial diagnosis of gallstone pancreatitis, an ASA score of three or four, and in emergency operations [39]. Amongst these factors, the non-elective status is the only controllable element that contributes to the length of stay. For this reason, we chose to analyze this factor and defined length of hospitalization as the number of days in the hospital from the operative end till the day of discharge. From our study, the length of hospitalization was not significantly prolonged by performing IOCs in an elective setting (p=0.085), whether the IOCs were straightforward or difficult to interpret as compared to an emergency setting (p=0.003). However, in emergencies, confounding factors such as the duration of intravenous antibiotics, concomitant lung infection, and sepsis naturally warrant a longer duration of treatment hence prolonging the duration of hospitalization.

Another clinical component we looked at was the effect of IOCs on the duration of surgery. The duration of IOC ranges from 4.3 to 18 minutes and timing varies based on technique, experience, and hospital resources [40], [41], [42]. From our study, performing IOCs in a daycare, elective, or emergency setting, did not significantly prolong the duration of surgery (p=0.069, p=0.478, p=0.471 respectively). These important clinical components (length of hospitalization and duration of surgery) have an overall impact in terms of reducing financial burdens and bed availability pressures on the hospital. This is particularly important for the bed occupancy rates (BOR) in tertiary government hospitals in Malaysia as they are usually at 75–100 % of their bed capacity.

Long waiting lists for elective cholecystectomies have been a sign of the inefficiency of medical services worldwide, particularly in government-funded hospitals. This problem is not foreign to our local setting due to limited operating theatre resources. To exacerbate this limitation, the benign nature of gallstone disease has led to an unavoidable postponement of elective cholecystectomies to make way for malignant cases to be operated on early. Early emergency cholecystectomy (less than 72 h) is currently the recommended management for acute cholecystitis [43, 44] whilst the waiting time for elective cholecystectomy of less than 30 days was recommended to reduce recurrent biliary colic, cholecystitis, cholangitis, gallstone pancreatitis, and revisits to the emergency department [45]. In our study, the average waiting time from diagnosis to elective cholecystectomy was 4.3 months. This time frame is significant, as the longer the waiting time is for elective cholecystectomy, the higher the likelihood of developing complications from simple cholelithiasis such as CBD stones and cholangitis, as it allows more time for CBD stones to pass before cholecystectomy. As the waiting time of 4.3 months is much longer than the recommended 30 days for elective cholecystectomy, performing routine IOC in our clinical setting is justifiable to detect these CBD stones.

### Strengths and limitations

Unlike most studies in the literature, this study focused on patients with normal LFTs. This is important because it is in this group of patients that CBD stones are undetected and not treated. This study also demonstrated the use of serum ALP and GGT in combination, as an affordable method in screening for stones post cholecystectomy. Although the study was performed in a high-volume centre and was prospective in nature, the study duration of 15 months contributes to a relatively small sample size as compared to numbers produced from retrospective population-based studies. The duration of the study limits the duration of monitoring which may not be adequate to assess for future development of CBD stones or hepatolithiasis as well as readmissions related to these stones. However, this follow-up duration of 3 months does not deviate from our normal practice for patients who have undergone simple cholecystectomies. It would be ideal to mimic the usual local clinical practice as much as possible to minimize the cost incurred to the patients and hospital. Being a single cohort study of patients who underwent cholecystectomy with IOC, this limited our ability to compare with a cohort of patients who underwent cholecystectomy without IOCs. The operating surgeons are comprised of different levels of experience; this could lead to biases in terms of their ability to interpret IOCs. Although a combination of serum ALP and GGT was used for its reliability and cost-effectiveness, other potential factors may cause a raised ALP and GGT which needs to be considered such as post-operative stricture, drug-induced, and infiltrative diseases of the liver [46].

### Future direction

The results of this study could be further strengthened by increasing the duration of study, as 80 % of patients with retained stones remain asymptomatic hence undetected at least 4 years before their presentation [47]. This study can be extrapolated by comparing other methods of detecting CBD stones such as fluorescent cholangiography with indocyanine green (ICG) or laparoscopic CBD exploration. This prospective study could potentially pioneer future local studies relating to IOCs, as many published data are retrospective in nature. A comparison between cohorts of ‘with IOC’ and ‘without IOC’ can be performed to compare its clinical outcomes in terms of duration of surgery and length of stay. The results from this study on difficult IOC interpretation could be used to augment training in cholangiography interpretation as this method has proven to be useful whether done routinely or selectively by general surgeons or HPB surgeons. As cost contributes significantly to decision-making in most government-funded hospitals, a cost-effective analysis should be conducted to further evaluate the health economic impact of IOCs in our local setting.

## Conclusions

We have demonstrated that routine IOC is a specific diagnostic tool with a good negative predictive value that is useful to exclude the presence of CBD stones when LFT is normal. Whether it is performed in an elective or emergency setting, IOCs do not significantly prolong the length of hospitalization or duration of the cholecystectomy, hence reducing the incidence of undetected retained stones and preventing its complications effectively.

## Supplementary Material

Supplementary Material
