# Supplementary material for: Is there a role for routine intraoperative cholangiogram in diagnosing CBD stones in patients with normal liver function tests? A prospective study
Source: Innov Surg Sci. 2024 Mar 8;9(1):37–45. doi: 10.1515/iss-2023-0059 (PMC11138406; doi:10.1515/iss-2023-0059)
Supplement: Supplementary file 1 — Supplementary Material [file j_iss-2023-0059_suppl_001.pdf]

Reviewer Assessment

YP Lim et al.: Is There A Role For Routine Intraoperative Cholangiogram In Diagnosing CBD Stones In Patients With Normal Liver Function Tests? – A Prospective Study

Reviewers' Comments to Original Submission

Reviewer 1: Wong, Wei Jin

Date received: 14-Dec-2023
Reviewer recommendation: **Return to author for major modifications**
Reviewer overall scoring: **Medium**

Comments to author:
Thank you for inviting me to review this article. The authors have attempted to answer the question of whether routine intra-operative cholangiogram is required for patients with normal biochemical markers, going for laparoscopic cholecystectomy. This is a topic which is still controversial, therefore, this article attempting to add to the available literature on the topic is valuable indeed. However, the article requires much refinement, as the justification of the study is not well defended, and the discussion points are not well argued.

Individual comments are as below:
Abstract:
The importance of the work needs to be further emphasized in the introduction, the authors should emphasize the clinical impact of missed common bile duct stones.
The methodology should be more succinct, to increase readability.

Main article:
Introduction: in general, the introduction remains unfocused and the importance of the study is not emphasized. The authors should defend why positive IOCs needed to be confirmed with ERCPs, as this subjects the patients to two procedures (and ERCP is not without risk). If the IOC was positive, would this not already answer the question of whether IOC is needed in normal liver function tests?
Page 2, line 54: this reviewer disagrees with the statement that "cholelithiasis is one of the most difficult diseases to treat". It is a common disease, and the treatment algorithms for it are well-established in surgical postgraduate curriculums.
Page 3, line 10: why does the use of IOC remain controversial? Kindly expand on this point.
Page 3, line 15: is the author referring to CBD stones detected on IOC? Or in the general population? Subsequently, what is the incidence of cystic stump leak post cholecystectomy in patients with retained CBD stones?
Page 3, line 24 – 32: this reviewer is unsure as to the utility of this paragraph. It does not defend the need for this study, perhaps this paragraph needs to be edited or deleted.

Methodology:
Page 3, line 44: was this study randomized? How did the authors decide on the timeline of 15 months? Also, this reviewer suggests that the inclusion and exclusion criteria be presented in the flow diagram, to increase readability. The authors should also include what the pre-set p-value is.
Page 3, line 55: "cholecystectomies were excluded from this study". Was this perhaps a typo? As the study includes patients who had cholecystectomies.
Page 4, line 13: as mentioned, why does IOC need to be validated with ERCP? This is not in congruence with the research question posed.
Page 4, line 29: "as quoted from previous study" – the grammar needs to be corrected.
Page 4, line 36 – 45: the calculations listed here would be better presented as formulas, and not text. This would enhance readability.
Page 4, line 47: please correct the grammar, Chi-squared and Mann-Whitney-U

Results:
Page 5, line 5 – 9: the reasons for exclusion can be listed in the flow diagram, removing the need for these sentences.
Page 5, line 12: the grammar should be improved here.

Page 5, line 32 – 35: the authors state that there is no difference in surgical time and length of stay in hospital, however this reviewer critiques the utility of this table, as it does not add much to the discussion. Should the authors choose the leave this in, the table should be presented in a manner that is more readable.

Discussion: why were the stones that were detected during IOC, not removed during the index procedure?
Page 6, line 14: the grammar needs to be improved
Page 6, line 14 – 45: this reviewer understands what the author is trying to convey, that IOC is equal to MRCP. However, the manner in which this is discussed needs to be improved. The flow of thought of the discussion is not sequential, and the author does not defend their case well enough.
Similarly, the discussion regarding ALP and GGT follows the same trend of being non-sequential and the author does not defend their case well enough.
Page 6, line 55: the detection rate of 3.3% cannot be concluded to be an accurate test. The incidence of retained stones of 2.3 – 5% is decided based on a common denominator. It is inaccurate to draw the conclusion that the detection rate is good as it is equal to the rate of retained stones.
Page 7, line 7 – 17: the utility of this discussion is debatable. An additional, low-risk procedure such as IOC should not contribute to length of stay. This reviewer suggests to remove this portion of the discussion altogether.
Page 7, line 19 – 27: the authors claim that the duration of surgery is not affected, however, in table (2), there seems to be significant discrepancy in the duration of surgery. How the authors calculated the statistical significance is questionable. A better way to present the data would be the present the mean or median surgical time. Perhaps this would better demonstrate the lack of statistical difference.
Page 7, line 40 – 42: the wait time of 4.3 months does not justify the need for IOC. The authors should elaborate more on why they think waiting time for surgery justifies the need for IOC..

Reviewer 2: anonymous

Date received: 26-Dec-2023
Reviewer recommendation: **Return to author for minor modifications**
Reviewer overall scoring: **High**

Comments to author:
The manuscript entitled "Is there a role for routine intraoperative cholangiogram in diagnosing CBD stones in patients with normal liver function tests? – A prospective study" addresses an issue that still under debate among surgeons performing cholecystectomy. In this study, patients with normal liver function tests underwent routine intraoperative cholangiography (IOC). Those with filling defects were subjected to ERCP. The authors conclude that IOC is useful to exclude CBD stones in case of normal liver function parameters without prolonging the hospitalization and the duration of cholecystectomy.
The study is well written and concise. It adds further evidence to an issue that is currently under debate. Only minor points might be addressed:
- Please define abbreviations upon first use, e.g. ASGE and USG.
- A significant number of patients undergoes ERCP based on IOC without CBD stone being detected upon ERCP. Particularly in younger patients this is an additional exposure to irradiation without benefit. This should be discussed.
- A major issue of the study is the lack of a control group and the short follow-up period. This is partly discussed in the discussion section but should be extended.

Authors' Response to Reviewer Comments

Date received: 09-Jan-2023

Reviewer 1:

Abstract:
The importance of the work needs to be further emphasized in the introduction, the authors should emphasize the clinical impact of missed common bile duct stones.
Corrected in manuscript

The methodology should be more succinct, to increase readability.
Corrected in manuscript

Main article:
Introduction: in general, the introduction remains unfocused and the importance of the study is not emphasized. The authors should defend why positive IOCs needed to be confirmed with ERCPs, as this subjects the patients to two procedures (and ERCP is not without risk). If the IOC was positive, would this not already answer the question of whether IOC is needed in normal liver function tests?

The reason why positive IOC requires an ERCP is to first, confirm the presence of CBD stones that were detected on IOC. Secondly, with ERCP therapeutic intervention can be performed eg. Stone clearance or stenting if it is required. The authors agree that ERCP is invasive and has its set of complications but it is less invasive as compared to an open CBDE. If IOC was positive, but the ERCP is negative (false negative) which means there were no stones present in the first place for patients with normal LFTs and no need for IOC in the first place.

Page 2, line 54: this reviewer disagrees with the statement that "cholelithiasis is one of the most difficult diseases to treat". It is a common disease, and the treatment algorithms for it are well-established in surgical postgraduate curriculums.
Corrected in manuscript

Page 3, line 10: why does the use of IOC remain controversial? Kindly expand on this point.
Corrected in manuscript

Page 3, line 15: is the author referring to CBD stones detected on IOC? Or in the general population?
Corrected in manuscript

Subsequently, what is the incidence of cystic stump leak post cholecystectomy in patients with retained CBD stones?
The incidence of bile leak post cholecystectomy is between 1.4-7%, and the most common cause is a cystic stump leak. An estimate of 20-35% of cystic stump leaks are caused by retained CBD stones due to an increased in intraluminal pressure.
This was added to the manuscript

Page 3, line 24 – 32: this reviewer is unsure as to the utility of this paragraph. It does not defend the need for this study, perhaps this paragraph needs to be edited or deleted.
This paragraph is to define the definition of high risk and low risk patients, as our study in focused on low risk patients. It does not defend the need for this study, however it is required in terms of defining these group of patients that we are focusing on.

Methodology:
Page 3, line 44: was this study randomized? How did the authors decide on the timeline of 15 months? Also, this reviewer suggests that the inclusion and exclusion criteria be presented in the flow diagram, to increase readability. The authors should also include what the pre-set p-value is.
This study was not randomised as all patients who underwent cholecystectomy and IOC and fulfilled the inclusion criteria were included.The timeline of 15 months was determined after achieving the adequate sample size of 180 patients.

Page 3, line 55: "cholecystectomies were excluded from this study". Was this perhaps a typo? As the study includes patients who had cholecystectomies.
Corrected in manuscript

Page 4, line 13: as mentioned, why does IOC need to be validated with ERCP? This is not in congruence with the research question posed.
As mentioned above, the ERCP is to confirm the findings of the IOC and allows for therapeutic intervention.

Page 4, line 29: "as quoted from previous study" – the grammar needs to be corrected.
Corrected in manuscript

Page 4, line 36 – 45: the calculations listed here would be better presented as formulas, and not text. This would enhance readability.
Corrected in manuscript

Page 4, line 47: please correct the grammar, Chi-squared and Mann-Whitney-U
Corrected in manuscript

Results:
Page 5, line 5 – 9: the reasons for exclusion can be listed in the flow diagram, removing the need for these sentences.
Corrected in manuscript

Page 5, line 12: the grammar should be improved here.
Corrected in manuscript

Page 5, line 32 – 35: the authors state that there is no difference in surgical time and length of stay in hospital, however this reviewer critiques the utility of this table, as it does not add much to the discussion. Should the authors choose the leave this in, the table should be presented in a manner that is more readable.
The authors plan to keep this table as it is part of our secondary objective; which is to look at the clinical impact of routine IOC in terms of duration of surgery and length of stay. We have edited the table so that it is more readable.

Discussion: why were the stones that were detected during IOC, not removed during the index procedure?
If stones were present, they were removed during a postoperative ERCP which was done a day after the cholecystectomy in the fluoroscopy suite. In our clinical setting, as with most tertiary hospitals in Malaysia, there is no luxury of operative time especially during the elective OTs to proceed with an immediate ERCP on table. As mentioned before, ERCP is less invasive as compared to CBDE, hence a post-operative ERCP is more suited to our clinical setting.

Page 6, line 14: the grammar needs to be improved
Corrected in manuscript

Page 6, line 14 – 45: this reviewer understands what the author is trying to convey, that IOC is equal to MRCP. However, the manner in which this is discussed needs to be improved. The flow of thought of the discussion is not sequential, and the author does not defend their case well enough.
Corrected in manuscript

Similarly, the discussion regarding ALP and GGT follows the same trend of being non-sequential and the author does not defend their case well enough.
Corrected in manuscript

Page 6, line 55: the detection rate of 3.3% cannot be concluded to be an accurate test. The incidence of retained stones of 2.3 – 5% is decided based on a common denominator. It is inaccurate to draw the conclusion that the detection rate is good as it is equal to the rate of retained stones.
Corrected in manuscript

Page 7, line 7 – 17: the utility of this discussion is debatable. An additional, low-risk procedure such as IOC should not contribute to length of stay. This reviewer suggests to remove this portion of the discussion altogether.
The authors have decided to keep this discussion as it adds to the safety value and discussion of performing routine IOCs. As this is also part of the secondary objectives looking at clinical impact (length of stay and duration of surgery), the discussion on this matter needs to be done.

Page 7, line 19 – 27: the authors claim that the duration of surgery is not affected, however, in table (2), there seems to be significant discrepancy in the duration of surgery. How the authors calculated the statistical significance is questionable. A better way to present the data would be the present the mean or median surgical time. Perhaps this would better demonstrate the lack of statistical difference.
This significant discrepancy could be attributed to the numbers in each group (daycare, elective, emergency) as they are all not equal.

Page 7, line 40 – 42: the wait time of 4.3 months does not justify the need for IOC. The authors should elaborate more on why they think waiting time for surgery justifies the need for IOC.
The longer the waiting time is for surgery, the higher the likelihood of developing complications from simple cholelithiasis such as CBD stones, cholangitis, cholecystitis and pancreatitis. This would require an IOC to evaluate the biliary anatomy, more complex treatments and further delay in the cholecystectomy to solve the primary problem should ERCP and stenting be required for the acute problem.

Reviewer 2:

The The manuscript entitled "Is there a role for routine intraoperative cholangiogram in diagnosing CBD stones in patients with normal liver function tests? – A prospective study" addresses an issue that still under debate among surgeons performing cholecystectomy. In this study, patients with normal liver function tests underwent routine intraoperative cholangiography (IOC). Those with filling defects were subjected to ERCP. The authors conclude that IOC is useful to exclude CBD stones in case of normal liver function parameters without prolonging the hospitalization and the duration of cholecystectomy.
The study is well written and concise. It adds further evidence to an issue that is currently under debate. Only minor points might be addressed:
Please define abbreviations upon first use, e.g. ASGE and USG.
Corrected in manuscript

A significant number of patients undergoes ERCP based on IOC without CBD stone being detected upon ERCP. Particularly in younger patients this is an additional exposure to irradiation without benefit. This should be discussed.
Corrected in manuscript under Discussion

A major issue of the study is the lack of a control group and the short follow-up period. This is partly discussed in the discussion section but should be extended.

This study was a single cohort study and not an RCT hence there was no control group. The study can be expanded by incorporating a control group or performing an RCT in the future.
The patients were reviewed 2 weeks post operatively and another time 3 months post operatively to review the histopathological findings of the cholecystectomy specimen. (This has been added to the methodology) However, we acknowledge that this is a rather short duration for further stones to develop. We used the duration of 3 months as in our clinical practice, most uncomplicated cholecystectomies are seen at 3 months post operatively and subsequently discharged if they are well.

Reviewers' Comments to Revised Submission

Reviewer 1: anonymous

Date received: 31-Jan-2024
Reviewer recommendation: **Return to author for minor modifications**
Reviewer overall scoring: **High**

Comments to author:

The authors The authors have submitted a revision of a previously-reviewed article entitled "Is There A Role For Routine Intraoperative Cholangiogram In Diagnosing CBD Stones In Patients With Normal Liver Function Tests? – A Prospective Study"
It attempts to answer a controversial topic regarding the treatment of cholelithiasis and choledocolithaisis, in particular, whether or not an additional procedure during cholecystectomy (IOC) reduces the risk of symptomatic choledocolithaisis after index cholecystectomy, by detecting asymptomatic choledocolithaisis.
In general, the article has addressed the comments from the previous reviewer.
The article is generally well-written, with a convincing argument and an adequate discussion.
The abstract is succinct and the reader gets an overall understanding of the article from it.
However, the introduction should also include that IOC is a purely diagnostic procedure, thus the need for ERCP after IOC for therapeutic purposes. The reasons that IOC requires validation by ERCP should also be explained – is this because IOC is deemed to be less accurate than ERCP? Or that the interpreting clinicians are not as skilled at interpreting IOC compared to ERCP? Whatever the reason is, it should be explained.
To demonstrate that there is no difference between length of stay with and without IOC, Table 2 should compare these 2 variables. The utility of just "daycare" compared with "elective" and "emergency" is limited. Duration of surgery should be displayed as the mean, in minutes, not as an ordinal scale. So table 2 should be redone to show these differences – with and without IOC, and displayed as mean/median rather than ordinal data.
Also, there are a few grammatical errors that should be corrected before publication can be made.
Should these corrections be made, I strongly recommend this article for publication

Reviewer 2: anonymous

Date received: 19-Jan-2024
Reviewer recommendation: **Accept in present form**
Reviewer overall scoring: **High**

Comments to author:

My questions are answered

Authors' Response to Reviewer Comments on Revised Submission

Date received: 01-Feb-2024

Reviewer 1

In general, the article has addressed the comments from the previous reviewer. The article is generally well-written, with a convincing argument and an adequate discussion. The abstract is succinct and the reader gets an overall understanding of the

article from it. However, the introduction should also include that IOC is a purely diagnostic procedure, thus the need for ERCP after IOC for therapeutic purposes.
Corrected in manuscript

The reasons that IOC requires validation by ERCP should also be explained – is this because IOC is deemed to be less accurate than ERCP? Or that the interpreting clinicians are not as skilled at interpreting IOC compared to ERCP? Whatever the reason is, it should be explained.
Corrected in manuscript
The purpose of performing an ERCP after the IOC is to confirm the presence of stones by firstly, re-interpreting the cholangiogram during the ERCP. Secondly, by therapeutically removing the detected stone by trawling during ERCP, this objectively confirms the presence or absence of CBD stones seen during IOC.

To demonstrate that there is no difference between length of stay with and without IOC, Table 2 should compare these 2 variables. The utility of just “daycare” compared with “elective” and “emergency” is limited.
As the inclusion criteria for this study is ‘all patients who underwent cholecystectomy and IOC’, there were no patients in this cohort that were ‘without IOC’ as they were excluded. Hence, the comparison cannot be made. However, the authors acknowledge that this is a limitations of the study and future studies on this comparison can be done (added in manuscript). From this study, the only comparison we were able to make was those who ,underwent cholecystectomy and IOCs in the 3 different settings; daycare, elective and emergency.

Duration of surgery should be displayed as the mean, in minutes, not as an ordinal scale. So table 2 should be redone to show these differences – with and without IOC, and displayed as mean/median rather than ordinal data.
As mentioned above, as the inclusion criteria for this study is ‘all patients who underwent cholecystectomy and IOC’, there were no patients in this study that were ‘without IOC’ as they were excluded. Hence, the comparison cannot be made.
The Duration of Surgery results were recalculated and changed to means as suggested by the reviewer.
Corrected in manuscript

Also, there are a few grammatical errors that should be corrected before publication can be made.
Corrected in manuscript

Comments by the Editorial Office to the Editor-in-Chief Decision

All reviewer comments were addressed adequately and the manuscript should be published in its present stage.
